# Prenatal cell‐free DNA screening for fetal aneuploidy in pregnant women at average or high risk: Results from a large US clinical laboratory

**DOI:** 10.1002/mgg3.545

**Published:** 2019-01-31

**Authors:** Carrie Guy, Farnoosh Haji‐Sheikhi, Charles M. Rowland, Ben Anderson, Renius Owen, Felicitas L. Lacbawan, Damian P. Alagia

**Affiliations:** ^1^ Quest Diagnostics Nichols Institute San Juan Capistrano California

**Keywords:** cfDNA prenatal screening assay, fetal aneuploidy, genetic counseling, microdeletion, microduplication, positive predictive value, sex chromosome aneuploidy, trisomy 13, trisomy 18, trisomy 21

## Abstract

**Background:**

We evaluated the performance of a cell‐free DNA (cfDNA) prenatal screening assay for trisomies 21, 18, and 13, and sex chromosome aneuploidies (SCAs) among a population of pregnant women that included both those at average and high risk.

**Methods:**

Specimen collection, cfDNA extraction, massively parallel sequencing, and bioinformatics analysis were conducted per laboratory protocol. Assay results, concordance with pregnancy outcomes, and performance characteristics were evaluated.

**Results:**

A total 75,658 specimens from 72,176 individual pregnant women were received. Technical reasons accounted for 288 (0.4% of all received samples) tests not performed. In the final analysis cohort (*N* = 69,794), 13% of pregnancies were considered at average risk and 87% at high risk. Mean gestational age at specimen collection was 15.1 weeks. Of the 69,794 unique pregnancies, 1,359 (1.9%) had positive test results. Among the results with confirmed outcomes, PPV for trisomies 21, 18, and 13 was 98.1%, 88.2%, and 59.3%, respectively; the PPV was 69.0% for SCAs and 75.0% for microdeletions. Overall, PPV was 87.2%, sensitivity was 97.9%, and specificity was 99.9%.

**Conclusion:**

This cfDNA prenatal screening assay provides highly accurate discrimination between affected and unaffected pregnancies among a population of pregnant women at average and high risk for fetal genetic abnormalities.

## 1 BACKGROUND

Cell‐free DNA (cfDNA) from peripheral blood of pregnant women is increasingly used to screen for fetal chromosomal aneuploidies, including Down syndrome (trisomy 21), Edwards syndrome (trisomy 18), Patau syndrome (trisomy 13), and sex chromosome aneuploidies (SCAs) (Bianchi et al., 2014; Chiu et al., 2008; Ehlrich et al., 2011; Guex et al., 2013; Jiang et al., 2012; McCullough et al., 2014; Nicolaides, Syngelaki, Gil, Atanasova, & Markova, 2013; Norton et al., 2015; Palomaki et al., 2012; Porreco, Garite, Maruel, & Marusiak, 2014; Sparks, Struble, Wang, Song, & Oliphant, 2012; Strom, Anderson et al., 2017; Strom, Maxwell, & Owen, 2017; Taneja et al., 2016; Zhang et al., 2015). Recently, cfDNA assays have incorporated technological advancements, such as massively parallel sequencing, and studies have shown superior performance of these assays to traditional screening methods, as well as earlier developed assays for the detection of trisomies 21, 18, and 13 (Bianchi et al., 2014; Guex et al., 2013; Jiang et al., 2012; McCullough et al., 2014; Nicolaides et al., 2013; Norton et al., 2015; Porreco et al., 2014; Strom, Anderson et al., 2017; Strom, Maxwell et al., 2017; Taneja et al., 2016; Zhang et al., 2015).

The American College of Obstetricians and Gynecologists (ACOG) recommends aneuploidy screening or diagnostic testing for fetal genetic disorders for pregnant women of all ages (American College of Obstetricians & Gynecologists, 2007; Committee on Practice Bulletins‐Obstetrics, 2016). ACOG further notes that early studies have demonstrated similar cfDNA prenatal screening sensitivity and specificity in the general obstetric population and the high‐risk population. However, they note that the positive predictive value (PPV) would be expected to be lower in low‐risk populations due to the lower prevalence of aneuploidy in this group (Committee on Practice Bulletins‐Obstetrics, 2016). Based on the high positive predictive values (PPVs) of cfDNA prenatal screening for trisomies 21, 18, and 13, and other benefits of such screening assays (e.g., results received in early pregnancy), the American College of Medical Genetics and Genomics (ACMGG) recommends informing all pregnant women that noninvasive cfDNA prenatal screening assays are the most sensitive screening option for traditionally screened aneuploidies (Gregg et al., 2016). In addition, ACMGG recommends offering follow‐up genetic counseling and diagnostic testing when cfDNA prenatal screening yields positive results (Gregg et al., 2016).

The majority of studies evaluating the accuracy of cfDNA prenatal screening assays have been conducted on pregnant women at high risk for fetal aneuploidy pregnancy outcomes. However, two recent studies suggest the use of these assays in the US general population of pregnant women would provide prenatal healthcare benefits and be cost‐effective (Benn et al., 2015; Fairbrother, Burigo, Sharon, & Song, 2016). Furthermore, recent studies have found that technologically advanced cfDNA prenatal screening assays perform as consistently in the general population of pregnant women as in high‐risk populations (Norton et al., 2015; Taneja et al., 2016; Zhang et al., 2015). Further evaluation is warranted to better define the performance of cfDNA assays for routine use in general screening populations that include both average‐ and high‐risk pregnancies.

QNatal Advanced is a highly automated, laboratory‐developed test that uses a high‐yield method of cfDNA preparation, massively parallel sequencing, and a GC content correction algorithm (Strom, Anderson et al., 2017). Per protocol, karyograms are generated for results that initially indicate affected chromosomes, allowing for the prospective identification of maternal microduplications to reduce false‐positive rates (Strom, Anderson et al., 2017; Strom, Maxwell et al., 2017). The assay was introduced in the United States in 2015 by Quest Diagnostics. In an initial analysis of 31,278 clinical specimens from pregnant women at high risk for fetal aneuploidy, PPV for trisomy 21 (98%), trisomy 18 (92%), and trisomy 13 (69%) were higher than previously reported in other studies (Strom, Maxwell et al., 2017). To further assess the performance characteristics of QNatal Advanced in a larger population of pregnant women, we extended the evaluation of the assay to include both women at average and high risk for fetal genetic abnormalities.

## 2 PATIENTS AND METHODS

### 2.1 Patient population

Blood specimens were collected from pregnant women who consented to QNatal Advanced fetal aneuploidy testing at Quest Diagnostics as a part of routine medical care. The specimens studied included all specimens from the prior initial analysis of 31,278 clinical specimens from pregnant women at high risk for fetal aneuploidy (Strom, Maxwell et al., 2017).

### 2.2 Editorial policies and ethical considerations

The current study was a retrospective expanded analysis of collected data, which was anonymized and therefore considered exempt by the Western Institutional Review Board.

### 2.3 Specimen analysis

Specimen collection, cfDNA extraction, massively parallel sequencing, application of laboratory‐developed bioinformatics analysis pipeline, and scientific review and reporting of results were conducted as previously described by Strom, Anderson et al. (2017). During the study period, sequencing transitioned from being performed using the HiSeq 2500 system to the NextSeq 500 system by Illumina (San Diego, CA). Pooled libraries are loaded on a NextSeq 500 sequencing system (Illumina), where they undergo clonal amplification and sequencing by synthesis on a High Output flow cell. Our quality metrics require a minimum of 6 million mapped reads per patient specimen.

### 2.4 Tests not performed

Tests not performed (TNP) were categorized as pre‐analytic or post‐analytic. Pre‐analytic TNP included specimens from pregnancies with gestational age <10 weeks, specimens canceled per ordering provider request, and specimens with poor quality, collection error, or insufficient volume. Post‐analytic cancellations were considered as being related to either underlying biological or technical factors. Reasons related to underlying biological factors included low fetal fraction, repeat low fetal fraction, and uninformative DNA pattern. Technical reasons included quality metrics, laboratory processing issues, and TNP due to an unspecified reason.

### 2.5 Pregnancy characteristics

Pregnancy characteristic data, obtained at the time of specimen collection, included patient date of birth, gestational age at collection, and number of gestations. Pregnant women were considered at high risk if they met any of the following criteria: advanced age (≥35 years), an abnormal ultrasound and/or positive maternal serum screen (MSS) result, or a reported personal or family history of fetal aneuploidy. The indication of high‐risk factors was based on International Classification of Diseases (ICD‐9/ICD‐10) diagnosis codes and information reported to the laboratory by the ordering provider.

### 2.6 cfDNA prenatal screening assay results and pregnancy outcomes

Results were reported in a binary manner, either positive or negative. The total numbers and percentages of negative and positive assay results were determined. The distributions of positive test results for trisomies 21, 18, and 13, SCAs (45,X; 47,XXX; 47,XXY; 47,XYY), and microdeletions (22q; 15q; 11q; 8q; 5p; 4p; 1p36) were determined for the final analysis cohort. All positive test results were communicated to ordering providers; discussion of follow‐up testing options with ordering providers was conducted by Quest Diagnostics genetic counselors upon request. Pregnancy outcome information was obtained by either genetic counselors or genomics client services specialists. Confirmatory diagnostic testing by routine cytogenetic or microarray analysis was performed by our laboratory or was reported by ordering providers if performed elsewhere. An internal database of pregnancy outcomes is maintained and includes confirmatory diagnostic testing results, pregnancy outcomes, and reported abnormalities identified on ultrasound or physical examination.

### 2.7 Statistical analysis and determination of PPVs, sensitivity, and specificity

Descriptive statistics were utilized to describe the characteristics of the patient population (per unique pregnancy). Specimens for which tests were not performed due to either pre‐analytic or post‐analytic reasons were excluded from the analysis. In addition, specimens were excluded from the analysis if gestational age was not reported or was reported as <10 weeks or >42 weeks, or if patients’ ages at delivery were missing or reported as <13 years old. Among the remaining specimens, unique pregnancies were identified. The numbers of cfDNA prenatal screening results that were concordant and discordant with pregnancy outcomes were determined and PPVs and overall negative predictive value (NPV) were calculated. PPVs and sensitivity were estimated as TP/(TP+FP) and TP/(TP+FN), respectively, among the subset of specimens with confirmed outcomes. NPV and specificity were estimated as TN/(TN+FN) and TN/(TN+FP), respectively. When estimating NPV and specificity, we assumed true‐negative outcomes unless alerted otherwise by the ordering provider, since follow‐up is not typically performed on negative test results. Analyses were performed using the R statistical package (version 3.4.2) (R Core Team, 2017).

## 3 RESULTS

In all, 75,658 specimens from 72,176 individual pregnant women were received for testing.

### 3.1 Tests not performed

Of the total specimens received, 2,113 tests were canceled prior to analysis (pre‐analytic TNP) and 2,634 were canceled after analysis (post‐analytic TNP). Among the post‐analytic TNP, 2,346 (3.1% of total specimens received) were canceled for reasons related to underlying biological factors; 288 (0.4% of total specimens received) were canceled for technical reasons. Mean maternal age at collection for women with post‐analytic TNP specimens was 34.7 ± 5.4 years. The gestational age of post‐analytic TNP specimens was reported for 40 cases with mean 14.1 ± 3.6 weeks. From 2,634 post‐analytic TNP specimens, 79% had high‐risk indications; 67.3% were advanced maternal age, 7.4% had an abnormal ultrasound, 9% had a positive MSS, and 6.4% had a personal or family history of fetal aneuploidy. Further details of the TNP specimens are reported in Table 1.

**Table 1:** Summary of tests not performed (TNP)

TNP	*n*	% of TNP (*n* = 4,747)	% of total specimens (*n* = 75,658)
Pre‐analytic TNP	2,113	44.5	2.8
Gestational age <10 weeks	594	12.5	0.8
Test canceled per provider request	447	9.4	0.6
Specimen quality	718	15.1	0.9
Collection error	295	6.2	0.4
Insufficient specimen volume	59	1.2	0.07
Post‐analytic TNP	2,634	55.5	3.5
Underlying biological factors	2,346	89.1	3.1
Low fetal fraction	1,954	41.2	2.6
Repeat low fetal fraction	355	7.5	0.5
Uninformative DNA pattern	37	0.8	0.05
Technical	288	10.9	0.4
Quality metrics	232	4.9	0.3
Lab processing issue	25	0.5	0.03
TNP‐not specified	31	0.7	0.04

**Figure 1:**
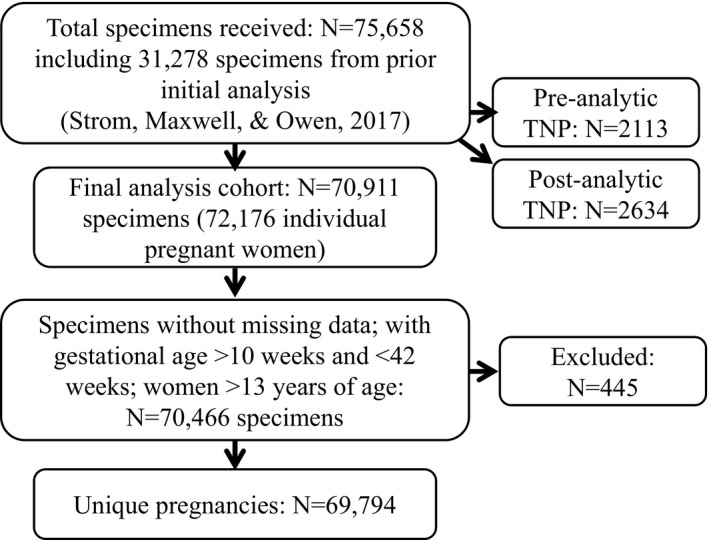
Flow of study specimens. TNP: test not performed

For the final analyses, all TNP samples were excluded. An additional 445 samples were excluded because the gestational age was not reported or was reported as <10 weeks or >42 weeks, or the patients’ ages at delivery were missing or reported as <13 years old. Of the remaining 70,466 specimens, 69,794 unique pregnancies were identified. The flow of study specimens is depicted in Figure 1.

### 3.2 Pregnancy characteristics

Characteristics of the 69,794 unique pregnancies are shown in Table 2. Of the 69,441 individual pregnant women, 69,088 (99.5%) submitted specimens from only one pregnancy and 353 (0.5%) submitted specimens from two unique pregnancies. Mean maternal age at delivery was 35.2 ± 5.8 years; 69% of the pregnant women were ≥35 years of age. The mean gestational age at specimen collection was 15.1 ± 4.9 weeks. Most specimens were collected in the 1st trimester (58.3%), followed by 2nd trimester (38.8%), and 3rd trimester (2.9%). Twin gestations accounted for 2% (*N* = 1,388) of specimens, and higher order multiple gestations accounted for 0.02% of specimens (*n* = 13).

**Table 2:** Pregnancy characteristics

	Final analysis cohort
	*N*_preg_ = 69,794
Number of pregnancies	
*N*_patient_	69,441
Initial pregnancy	69,088 (99.5%)
Initial and second pregnancy	353 (0.5%)
Maternal age at delivery^a^	
Mean ± *SD*	35.2 ± 5.8
<35 years old	21,792 (31%)
≥35 years old	48,002 (69%)
Gestational age (weeks)	
Mean ± *SD*	15.1 ± 4.9
1st trimester (10–13 weeks)	40,720 (58.3%)
2nd trimester (14–27 weeks)	27,075 (38.8%)
3rd trimester (≥28 weeks)	1,999 (2.9%)
Multiple gestations	
Twins	1,388 (2%)
>2 fetuses	13 (0.02%)
High‐risk factors	
*N*_preg_	60,792 (87%)
Advanced age^b^	48,185 (79.2%)
Abnormal ultrasound	7,735 (12.7%)
Positive maternal serum screen	6,255 (10.3%)
Personal or family history	4,313 (7.1%)

a Age of patients with >1 pregnancy was considered for both pregnancies.

b 81 patients with advanced age were <32 years old and had a diagnosis code for advanced age.

Of the 69,794 pregnancies, 8,949 (13%) did not have an indication for high risk for fetal aneuploidy and thus were considered to be at average risk; 60,792 (87%) were at high risk for fetal aneuploidy. Of the high‐risk pregnancies, 79.2% were advanced maternal age, 12.7% had an abnormal ultrasound, 10.3% had a positive MSS, and 7.1% had a personal or family history of fetal aneuploidy.

### 3.3 Positive test results

In the final analysis cohort (*n* = 69,794), 1,359 specimens had positive results (1.9%), of which 725 (53.3%) were positive for trisomy 21 (1.04% of final analysis cohort), 215 (15.8%) were positive for trisomy 18 (0.31% of final analysis cohort), and 140 (10.3%) were positive for trisomy 13 (0.20% of final analysis cohort) (Table 3). Of the specimens with positive test results, 253 (18.6%) were positive for SCAs (0.36% of final analysis cohort) (Table 3). The most frequent SCA was 45,X, which accounted for 8.3% (*n* = 113) of all positive results, followed by 47,XXY (5.0% [*n* = 68] of all positive results), 47,XXX (3.2% [*n* = 43] of all positive results), and 47,XYY (2.1% [*n* = 29] of all positive results). Of the positive test results, 26 (2.0%) were positive for microdeletions (0.04% of final analysis cohort) (Table 3). The most prevalent microdeletion detected was 22q (1.0% of all positive results).

**Table 3:** Incidence of positive results, outcomes obtained, and positive predictive values (Total *N* = 69,794 pregnancies)

	Trisomy 21	Trisomy 18	Trisomy 13	SCAs	Microdeletions	Overall
Positive results	725	215	140	253	26	1,359
Incidence^a^	1.04%	0.31%	0.20%	0.36%	0.04%	1.95%
Outcomes obtained	256	93	59	58	12	478
Concordant^b^	251 [6]	82 [26]	35 [6]	40 [10]	9 [1]	417
Discordant	5	11	24	18	3	61
PPV: confirmed outcomes	98.1%	88.2%	59.3%	69.0%	75.0%	87.2%
CI	96–99	80–93	47–71	56–79	47–91	84–90

Note

CI: confidence interval; PPV: positive predictive value; SCA: sex chromosome aneuploidies.

a Incidence: The proportion of affected pregnancies.

b Concordant results are presented as the number of total outcomes confirmed [*N* without karyotype in parentheses].

### 3.4 PPVs, sensitivity, and specificity

Among all specimens with positive cfDNA results and outcome data, 245 cases of trisomy 21 (60.3%), 56 of trisomy 18 (13.8%), and 29 of trisomy 13 (7.1%) positive assay results were confirmed by karyotyping. Of the positive results in the final analysis cohort (*n* = 1,359), 61 (4.5%) had diagnostic testing (i.e., prenatal diagnosis or confirmatory cytogenetic testing after birth) that was discordant with the cfDNA prenatal screening result. Thus, the overall observed PPV for this cfDNA prenatal screening assay was 87.2% (417/478) among pregnancies with confirmed outcomes. Of specimens with negative results in the final analysis cohort, 9 (<0.1%) had diagnostic testing that was discordant with the cfDNA prenatal screening result. The overall NPV was thus 99.9% (68,426/68,435). Among the pregnancies with confirmed outcomes, PPVs were 98.1% for trisomy 21, 88.2% for trisomy 18, 59.3% for trisomy 13, 69.0% for SCAs, and 75.0% for microdeletions (Table 3). For the final analysis cohort, the total number of true‐positive results was 1,298 of 1,359 observed positive results; the number of false negatives was 9, for an overall sensitivity of 99.3% (1,298/1,307). For only results with confirmed pregnancy outcomes, the sensitivity was 97.9% (417/426). Of the total negative results (*n* = 68,435), 68,426 were true negative, for a specificity of 99.9% (68,426/68,487).

## 4 DISCUSSION

In this study, we assessed the performance of the QNatal Advanced cfDNA prenatal screening assay in a population that included pregnant women at average risk and pregnant women at high risk for fetal genetic abnormalities; for the outcomes obtained (*n* = 478; 35% of all positive test results), the assay yielded high PPVs for trisomies 21 (98.1%), 18 (88.2%), and 13 (59.3%); the PPV was 69.0% for SCAs and 75.0% for microdeletions. Additionally, the assay showed highly accurate discrimination between affected and unaffected pregnancies, with high sensitivity (97.9%) and specificity (99.9%). In this study population of pregnant women, 13% of the 69,794 pregnancies in the final analysis cohort were considered at average risk and 87% were at high risk for a pregnancy outcome of fetal aneuploidy. The high PPVs for trisomies in this study population were high, similar to previously reported PPVs for the assay among a population of high‐risk pregnant women (Strom, Maxwell et al., 2017).

Only 0.4% of the total specimens received had TNPs for technical reasons, but other reasons for post‐analytic TNPs include underlying biological factors (maternal fibroids, malignancy, fetal fraction), which can affect interpretation of cfDNA results. Reasons for low fetal fraction include fetal aneuploidy (Hui, 2016; Pergament et al., 2014) and high maternal body mass index (Hui, 2016; Livergood, LeChien, & Trudell, 2017); novel research has also found that maternal anticoagulant usage contributes to low fetal fraction levels (Grömminger et al., 2015; Hui, 2016; Wardrop et al., 2016). Another underlying biological factor may be maternal fibroids and malignancy, which have been associated with uninformative DNA patterns (Bianchi, Chudova et al., 2015). Identification of an uninformative DNA pattern prohibits the interpretation of the fetal result and thus results in a post‐analytic TNP. Since there is no current consensus regarding the management of uninformative DNA patterns identified in prenatal cfDNA screening these are not currently being reported. However, additional comment is provided on the report describing reason for the post‐analytic TNP. Additional research is needed in this area to continue to inform reporting practices. Collaboration with ordering providers and clinical outcome collection will provide additional insight in this area of research. Reporting practices continue to vary among clinical laboratories, despite emerging literature indicating the importance of measuring and communicating post‐analytic TNPs caused by underlying biological factors. This is reflected in the ACMGG guidelines related to cfDNA prenatal screening, which recommend discussion of diagnostic testing options after a TNP caused by low fetal fraction (Committee on Practice Bulletins‐Obstetrics, 2016). Therefore, fetal fraction has been included in this study to reflect the importance of transparent communication from laboratories to ordering providers to inform clinical management.

The PPVs reported in the current study are higher than most of those reported by prior studies that evaluated the use of cfDNA prenatal screening assays among populations that included high‐ and average‐risk pregnant women (Norton et al., 2015; Taneja et al., 2016; Zhang et al., 2015). The other studies reported PPVs in the following ranges: 80.9%–92.8% for trisomy 21, 74.3%–90.0% for trisomy 18, and 32.8%–50.0% for trisomy 13 (Norton et al., 2015; Taneja et al., 2016; Zhang et al., 2015). The higher PPVs in the current study may be explained by methodological differences between the QNatal Advanced assay and other assays, bioinformatics techniques, processes of scientific result review and reporting, and coordination of follow‐up studies. For example, the standard protocol for QNatal Advanced includes prospectively generating karyograms for positive test results to rule out false positives caused by maternal duplications (Strom, Maxwell et al., 2017). Additionally, genetic counselors support ordering providers to facilitate clinically appropriate follow‐up studies, which may improve the reporting of outcomes and lead to improved pregnancy management. As utilization of cfDNA screening by general practice providers increases, this expert resource supports busy practitioners with the most informative follow‐up testing options. Furthermore, the binary (positive/negative) reporting structure of the QNatal Advanced screening assay allows for clear communication of which pregnancies are at increased risk for fetal aneuploidy, thus avoiding confusion of what is the most clinically appropriate action related to “gray‐zone” or “suspected” results. Prior studies have demonstrated lower PPVs when “aneuploidy suspected” categories are utilized (Taneja et al., 2016).

We report a PPV of 69% (among confirmed outcomes) for SCAs with an incidence of 0.36% for SCA in >69,000 pregnant patients of both high‐ and average‐risk. In this study, prospective analysis to identify results suggestive of maternal SCA was conducted to avoid false‐positive results. This result review and reporting practice, combined with an integrated follow‐up testing and outcome program, may result in the high PPV for SCAs observed. Direct comparison of our results to previously published studies is difficult because study populations differ in size, demographics, and clinical characteristics. Peterson et al. (2017) reported an incidence of 19%, but the population was smaller (*n* = 712) and consisted only of high‐risk patients; the reported PPVs were broken down by SCA type and ranged from 26% (monosomy X) to 86% (47,XXY). In a study of 6,388 pregnancies, an SCA incidence of 0.83% was reported, with a PPV of 55% for monosomy X; demographics and clinical characteristics of the study population were not provided in the report (Pescia et al., 2017). Bianchi, Parsa et al. (2015) reported an SCA incidence of 1.1% among 18,161 specimens from women with a similar maternal age (mean age: 35.7 years) as our study population (mean age: 35.2 years); however, the PPV was not determined because of incomplete follow‐up. Additional research on the outcomes and recommended follow‐up of cfDNA screening for SCAs is warranted, as the most commonly identified SCA in this study (45,X) has established implications for pregnancy management related to the increased risk for cystic hygroma and congenital heart defect.

With our prenatal cfDNA screening assay, the overall PPV for microdeletions was 75.0%. This suggests practical clinical utility of this assay for the general obstetric population when performed in coordination with the advanced bioinformatics techniques, scientific result review, and reporting processes described here. Among the high‐risk population in the study of Peterson et al. (2017), PPVs for microdeletions ranged between 0% and 21%. The higher PPV demonstrated in this study may be attributed, in part, to the prospective analysis for maternal deletions, combined with strong assay performance and coordination of follow‐up by genetic counselors. At present, ACOG does not recommend routine screening for microdeletions, given the relatively limited research in this area and prior limited progress in their accurate detection (American College of Obsetricians & Gynecologist Committee on Genetics, 2015). This study contributes to the expanding literature in this area of clinical practice.

The main strength of this study is that it evaluated the performance of this cfDNA screening assay in a large population of pregnant women in the United States who were at average and high risk for fetal genetic abnormalities. The clinical landscape is evolving to reflect cfDNA screening as the recommended screen for fetal aneuploidy for all women. This study supports screening in women both at high‐ and average‐risk. The high PPVs for SCAs and microdeletions demonstrated in this study add to the limited body of literature on the performance of cfDNA for these conditions. This has implications for patients and providers who desire accurate screening options for these conditions.

One limitation of this study is the incomplete pregnancy outcome information, which is related to the reliance on provider reports in some cases. This limitation could have introduced bias into the performance evaluation, specifically into the calculation of negative predictive value. This demonstrates the importance of a coordinated effort between clinical laboratories and ordering providers in the collection of outcome data. Clinical laboratories are often dependent on outcome information shared by the ordering provider. Increased focus on the development of systematic outcome programs will enhance the reliability and quality of performance data. This also shows the value of genetic counselors coordinating follow‐up testing to guide clinically appropriate confirmation testing and following up to obtain pregnancy outcomes. Additional research on test utilization management by genetic counselors in this specialty may provide additional insight into the impact on performance evaluation data and clinically appropriate ordering. Another limitation of this study is that we included previously reported data on the high‐risk population of pregnant women (Strom, Anderson et al., 2017; Strom, Maxwell et al., 2017). Comparative analysis of the population of average‐risk and high‐risk patients was not conducted due to the small sample size of the average‐risk population. Additional research is warranted to further analyze the performance of prenatal cfDNA screening assays in the average‐risk population compared to the high‐risk population.

Despite the good performance of cfDNA prenatal screening assays in comparison to standard screening assays in having superior PPVs (Bianchi et al., 2014), they should remain a screening test and, as recommended by the ACMGG, abnormal results should be followed up with further prenatal diagnostic tests. Pre‐and post‐test education should be offered to all pregnant women to explain the potential expectations and limitations of the cfDNA prenatal screening assays. Furthermore, all patients with a positive result should be offered genetic counseling to discuss follow‐up diagnostic options.

In conclusion, we evaluated the performance of a cell‐free DNA (cfDNA) prenatal screening assay for trisomies 21, 18, and 13, microdeletions, and sex chromosome aneuploidies (SCAs) among a population that included both pregnant women at average risk and those at high risk. This analysis demonstrated high sensitivity, specificity, and PPVs for all conditions screened in the study population. The strong performance of this laboratory‐developed assay reflects advanced bioinformatics, expert scientific review, and specialized coordination of follow‐up studies.

## CONFLICT OF INTEREST

All authors are employees of Quest Diagnostics.
